# Community-based HyPertension Control (CHPC) in Nepal: Cluster randomized implementation trial protocol

**DOI:** 10.1016/j.puhip.2026.100772

**Published:** 2026-03-20

**Authors:** Archana Shrestha, Sujata Sapkota, Dinesh Timalsena, Pragya Sharma, Anna K. Porter, Drew B. Cameron, Ashley Hagaman, Dinesh Neupane, Meghnath Dhimal, Sushmita Mali, Lisasha Poudel, Reshu Agrawal Sagtani, Bandana Paneru, Pratiksha Paudel, Anjali Joshi, Dipak Raj Chaulagain, Robin Man Karmacharya, Donna Spiegelman

**Affiliations:** a Department of Public Health, Kathmandu University School of Medical Sciences, Dhulikhel, Nepal; b Institute for Implementation Science and Health, Kathmandu, Nepal; c Center for Methods in Implementation and Prevention Science, Yale School of Public Health, New Haven, CT, USA; d Department of Chronic Disease Epidemiology, Yale School of Public Health, New Haven, USA; e Department of Pharmacy, Manmohan Memorial Institute of Health Sciences, Kathmandu, Nepal; f Department of Health Policy and Management, Yale School of Public Health, New Haven, CT, USA; g Department of Social and Behavioral Sciences, Yale School of Public Health, New Haven, CT, USA; h Department of International Health, Johns Hopkins Bloomberg School of Public Health, Johns Hopkins University, Baltimore, MD, USA; i Nepal Health Research Council, Kathmandu, Nepal; j Department of Surgery (Cardio Thoracic and Vascular), Dhulikhel Hospital, Kathmandu University, Dhulikhel, Nepal; k Department of Biostatistics, Yale School of Public Health, New Haven, CT, USA; l Department of Public Health and Community Programs, Dhulikhel Hospital-Kathmandu University Hospital, Dhulikhel, Nepal; m NIHR Global Health Research Center for Multiple Long Term Conditions, Kathmandu, Nepal

**Keywords:** Hypertension, Female community health volunteers, Implementation trial, Nepal

## Abstract

**Objective:**

This study aims to evaluate the implementation i.e., reach, adoption, fidelity, and maintenance; and effectiveness on blood pressure reduction of implementation strategies to enhance uptake of community-based hypertension control intervention in Nepal.

**Study design:**

A cluster randomized, hybrid type III effectiveness-implementation trial.

**Methods:**

The study focuses on Reach, Effectiveness, Adoption, Implementation, and Maintenance of the RE-AIM framework implementation outcomes. We will conduct a cluster randomized trial in Sindhupalchowk district, randomizing 102 health facilities in the district into the CHPC intervention (n = 51) and standard care control (n = 51) groups. The CHPC includes community-based BP monitoring, lifestyle counseling and medication adherence monitoring. The implementation strategies of CHPC intervention will include a) leadership support and ongoing program promotion, b) capacity building of healthcare workers and Female Community Health Volunteers (FCHVs) in hypertension management, c) FCHV group meetings and home visits, and d) creating social networks to promote shared learning. Participants (n = 3,572, 35 in each of the 102 clusters) aged ≥30 years with BP ≥ 140/90 mmHg and/or on medications will be recruited. Participants from intervention clusters will be assessed against those residing in the control communities. Implementation outcomes (reach, adoption, implementation and maintenance) will be assessed using data from the process evaluation in intervention health facilities. The evaluation will use a mixed-method approach. For effectiveness, the mean decrease in systolic BP in the intervention group compared to the control group will be assessed 12 months after the intervention begins. The cost-effectiveness of the intervention will also be assessed. We will conduct observations, periodic reflections, focused group discussions and key informant interviews to explore implementation outcomes. Qualitative methods will be informed by the Consolidated Framework for Implementation Research (CFIR) and analyzed using a rapid qualitative analytic approach and thematic analysis.

**Discussion:**

This study will provide evidence for tested approaches and mechanisms for utilizing and embedding community health volunteers in hypertension care to mitigate hypertension burden and improve quality of life.

**Protocol version:**

2; October 2024.

**Trial registration:**

ClinicalTrials.gov NCT06081010*. Registered 12 October 2023,*https://clinicaltrials.gov/study/NCT06081010.

## 1 Introduction

Hypertension (HTN) is a significant global public health challenge, responsible for 13.5% of premature deaths, 54% of incident stroke, and 47% of Coronary Heart Disease (CHD) worldwide [1]. Of the estimated 1.2 billion adults with HTN globally, two-thirds live in low-and middle-income countries (LMICs) [2]. In Nepal, HTN affects 25% of adults [3], yet 44% remain unaware of their condition [4]. Among those diagnosed, only 33% receive treatment, and 12% achieve BP control [3,4].

Evidence-based interventions, such as weight loss [5], dietary modification [6], increased physical activity [7], lowered alcohol consumption [8] and consistent use of anti-HTN medications [9] remains the cornerstone of HTN prevention and control [10]. However, despite these interventions being proven effective, they have not yet been comprehensively translated into practice. Nepal adopted the Package of Essential Non-Communicable Diseases to strengthen HTN detection and management at primary healthcare facilities [11]. However, recent evaluations of PEN have identified multiple implementation barriers, including low perceived susceptibility, low health literacy, and misconceptions about HTN and its medications at individual level; peer pressure that supports physical inactivity and a high sodium diet at interpersonal level; norms supporting unhealthy eating and low medication adherence at community level; and unfilled human resource positions, overburdened healthcare staff, interrupted medical supplies and medicines, inefficient recording and reporting, and insufficient provider-patient interaction at health system level [[12], [13], [14], [15]].

To overcome these multi-level implementation barriers, we propose a community-based HTN management through FCHVs. FCHVs are trained community health volunteers [16], who have played critical roles improving maternal and child health in Nepal [17]. Emerging evidence suggests FCHV-led HTN control interventions can effectively improve hypertension outcomes, for example, a major trial in Nepal reported significant reductions in systolic and diastolic BP at one year among intervention participants as compared to controls (−4.90 mm Hg [−7.78, −2.00] and −2·63 mm Hg [−4·59 to −0·67], respectively) [18].

FCHVs led HTN managment is expected to address the implementation barriers in three ways. First, FCHVs will frequently engage with and educate patients with HTN, promoting their self-efficacy and empowering them to mitigate peer pressure and overcome negative cultural beliefs thus leading to a better quality of life. Second, FCHVs will improve health system efficiency by monitoring patient lifestyle behaviors, blood pressure, and medication adherence; and third, FCHVs will directly connect HTN patients with health care providers at health facilities through timely referrals.

### 1.1 Objectives

This study aims to implement and evaluate Community-based HyPertension Contro (CHPC) through FCHV mobilization within the framework of existing government healthcare systems. Specifically, we will **(a)** assess implementation outcomes of the CHPC at provider and health system levels, **(b)** assess its effectiveness compared to facility-based PEN on systolic blood pressure, and, **(c)** assess economic sustainability.

## 2 Methods

This protocol has been developed and reported following the Standard Protocol Items: Recommendations for Interventional Trials (SPIRIT) and Standards for Reporting Implementation Studies (StaRI) guidelines [19].

### 2.1 Study design

We will evaluate primarily Reach, Effectiveness, Adoption, Implementation and Maintenance (RE-AIM) framework [20] implementation outcomes and secondarily assess effectiveness outcomes. Primary implementation outcomes wil be measured in the intervention group. We will conduct a non-blinded two-arm cluster-randomized trial to assess the effectiveness of the intervention on mean systolic BP compared to the control group at 12 months. The health facility will be randomly assigned to the intervention or control group. The intervention arm will receive the community health worker (CHW) intervention and strategies, while the control arm will receive the standard of care.

### 2.2 Site

We will conduct the study in Sindhupalchowk district, one of the largest districts in Bagmati province, Nepal, consisting of nine rural and three urban municipalities (Fig. 2). The district spans hills to mountain region, where geography that facility-based healthcare delivery. This setting offer the opportunity to evaluate the intervention across diverse terrains and rural-urban contexts. The district lies within the catchment area of Dhulikhel Hospital-Kathmandu University School of Medical Sciences, which will directly support the program and facilitate participant referrals when needed.

### 2.3 Randomization

**Fig. 1:**
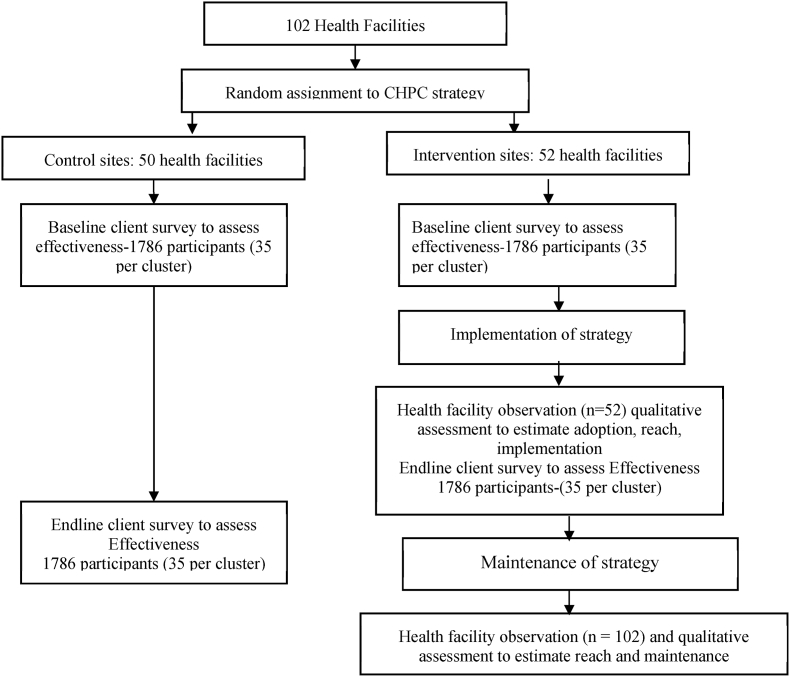
CHPC trial design.

A total of 102 health facilities will undergo randomization, stratified by municipality (Fig. 1). To ensure allocation concealment, the study not involved in recruitment will pre-generate 10 random allocation sequences for each of the 12 municipalities, using a computer-based random number generator. Each sequence will be placed inside an opaque, sequentially numbered, sealed envelope. During a public event, a neutral stakeholder, not affiliated with the research team, will select one sealed envelope, which will decide the final allocation. This process will prevent research team from predicting or influencing the assignment.

**Fig. 2:**
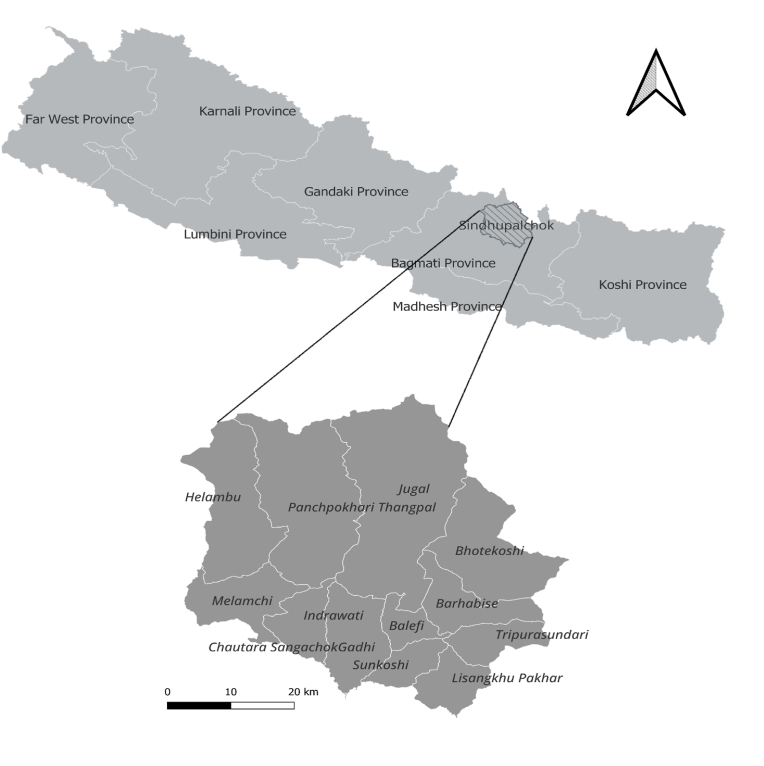
Study area, Sindhupalchowk District.

### 2.4 Study participants

#### 2.4.1 Health facilities and FCHVs

We will contact the health facilities assigned to the intervention group and invite them to participate in the study. FCHVs reporting to and providing services through these health facilities will be recruited with the support of the health facility in-charge.

#### 2.4.2 Participants with HTN

Individuals aged 30 years and older (based on Nepal's PEN protocol) [21] with high BP (≥140/90 mmHg) [22] residing in Sindhupalchowk will be eligible for inclusion. Individuals with severe illness requiring bed rest and pregnant women will be excluded. Community screenings will be conducted to identify the individuals eligible to participate. Trained research assistants (RAs) will explain the study objectives, procedures, voluntary participation, and the right to withdraw at any time to eligible participants. Written informed consent will be obtained before participation. For illiterate participants, verbal consent will be obtained, and a caregiver or witness will sign the consent form.

### 2.5 Intervention

The intervention involves task-shifting of HTN management roles to FCHVs, integrating the five components of the evidence-based interventions outlined in the PEN protocol-1 and 2 [21,23]:

(a) BP measurement and monitoring;
(b) Identify individuals with high BP and report to the health facility;
(c) Provide one-on-one counseling and lifestyle modification goal setting, including sodium reduction, healthy diet, alcohol and smoking abstinence, physical activity, and medication;
(d) Support medication adherence of HTN patients, delivered by the FCHVs trained for the intervention. The intervention will be conducted over a year and repeated during maintenance. The study is in the fourth quarter of the first year.

### 2.6 Implementation strategies

Implementation of the PEN interventions faces multilevel barrires: low literacy and HTN misconceptions amongst individuals, communitynorms promoting unhealthy lifestyles and improper medicine use, and health systems constrains such as limited and overburdened staff and supply interruptions, limited provider-patient interaction time and weak recording and reporting system [[12], [13], [14], [15]].To address these barriers, we have designed the following coordinated implementation strategies (Table 1):

a. **Leadership support, community engagement, and ongoing program promotion**: Ongoing support government leadership is critical to the successful implementation and sustainability of the CHPC. We wil form a steering committee consisting of representatives from central and provincial Ministries of Health (MoH), NCD experts, and health system experts. The committee will provide strategic guidance, oversee implementation, assess risks, and support sustainability planning. A Community Advisory Board, including people with HTN, community leaders, health staff, and political stakeholders will meet bi-to tri-annual basis. We will conduct project orientation at the province/district and municipal level, and health facility level. We will share the study progress in quarterly health system review meetings.
b. **Capacity building:** There is a growing recognition that capacity building can be enhanced by supporting training with ongoing coaching and technical assistance [[24], [25], [26]]. We will first conduct the training of the trainers (ToT), where at least one healthcare worker from each facility along with municipal health coordinators, will be trained as a trainer. The trained healthcare workers will then train FCHVs to implement the intervention. FCHV training will occur at the start and midpoint of implementation year. For both healthcare providers and FCHVs, the 3 day training will focus on three objectives: 1) knowledge of the PEN protocols 1 and 2, 2) skills to implement the intervention including communications, patient engagement, and monitoring progress [27], and 3) skills for barriers management [28]. Training for health care workers will also include skills development to train FCHV and provide ongoing coaching and technical assistance. All training will follow participatory adult learning principles [27,28]. Furthermore, FCHVs will receive flipchart, BP instruments, and recording forms, and reporting sheets.

**Table 1:** Implementation strategies’ core and adaptive components.

	Core components	Adaptive components
**Leadership support and ongoing program promotion**	Health Coordinators from the Provincial MoH will participate in the planning, execution and monitoring of the study. In provincial MoH health review meetings, hypertension-related indicators (proportion of population screened, HTN detected, HTN under medication, HTN control) will be discussed using the government's health management information system data.	Health coordinators will be encouraged to discuss how to engage patients and health workers in planning and monitoring CHPC. In review meetings, tip sheets will be provided listing the contextual challenges and possible solutions in achieving HTN screening, detection, medication, and control.
**Capacity building**	Healthcare workers training; FCHV training; Coaching and technical support from healthcare workers to FCHV in a monthly meeting	Health care workers can connect with the research staff on the telephone when needed; FCHVs can connect with healthcare workers on the phone when needed; FCHVs may be provided with the list and contact details of referral centers and related physicians to smoothen referral of patients with high BP
**FCHV Home visit and group meeting**	1st visit: introduction, rapport building, goal setting, BP monitoring; 2nd- 4th visit: BP monitoring, action and reflection, discussing problems faced in achieving their goals and implementing their action plans	Additional visits/meetings can be scheduled depending on patients' needs and FCHV's availability. May include family members who are decision makers during home visit session; May conduct the home visit session in groups living in the same household
**Social network**	Monthly meetings of health care workers in the municipality are held. Monthly meeting of FCHVs in the health facility.	May conduct experience sharing regarding the CHPC in meetings conducted for other purposes; May invite community leaders or patient representatives to the meetings

**Coaching and technical assistance**: We will provide ongoing support for FCHVs by training the healthcare workers to coach and supervise them. Research team members will attend the monthly meeting, offer phone support, and provide technical assistance. Healthcare workers will use monthly meetings and phone communication to support FCHVs. Furthermore, we will also connect intervention health facilities to the telemonitoring services of Dhulikhel hospital to support HTN case management and referrals, when needed.

c. **FCHV group meetings and home visits:** After completing training and competency assessment, FCHVs will conduct group meetings and home visits. From the list of patients with HTN enrolled at screening, FCHVs will form HTN patient group and organize four sessions in a year. They will first invite HTN patients to attend group meetings, those with uncontrolled HTN, adherence challenges, or inability to attend will receive home visits. In the first visit (∼ 90 min), FCHVs will explain the purpose, monitor blood pressure, discuss HTN and its consequences, and provide education using flip chart. Participants will choose lifestyle goals including physical activity, healthy diet, tobacco and harmful use of alcohol, attending medical follow-up, BP monitoring, and antihypertensive medication use. In subsequent sessions (∼ 60 min), the FCHVs will measure BP, review progress, address challenges, and update action plans with participants and their family members.
d. **Social network:** Using learning networks concept [29], we will facilitate information exchanges among healthcare workers and FCHVs to support shared problem-solving, learning and program implementation [30,31]. These exchanges will occur during regular municipal and health facility meetings. We will provide discussion questions, with healthworkers facilitating discussions.

### 2.7 Outcomes

Following the Hybrid Type III effectiveness-implementation appraoch, this study assesses both implementation and clinical outcomes (Table 2). The primary implementation: Reach, Adoption, Implementation, and Maintenance [20] in intervention arm, measured at health facility level. The clinical outcome is the mean change in systolic blood pressure (SBP) between intervention and control arms from baseline to 12 months, measured at the participant level. Secondary clinical outcomes diastolic BP, control, awareness of HTN diagnosis, lifestyle practices, medication adherence, quality of life, and economic costs compared between intervention and control groups.

**Table 2:** Study outcomes.

Implementation Outcomes			
		**Measures**	**Data Sources**
Reach	Percentage of FCHVs implementing the program participating in at least 8 of the 12 monthly meetings	At least 80% of the trained FCHVs will participate in at least 8 of the 12 monthly meetings;	Process tracking data, including the number of FCHVs participating in each group session meeting and themes discussed, as reported by the health facility in charge. Attendance records will be collected by study staff during monthly process tracking calls with the health facility in charge.
	Percentage of patients with hypertension in the community aware of their high BP status	At least 80% of the patients with hypertension in the community are aware of their diagnosis.	Participant survey
Effectiveness	Systolic BP (mmHg)	The net difference in mean systolic BP (mmHg) will be measured after 12 months between the intervention and control groups.	Baseline and end-line surveys
Adoption	Percentage of the health facilities intending to adopt the program.	Adoption will be a dichotomous measure (adopted vs not adopted). Adoption is defined as health facilities where FCHVs complete the initial FCHV training sessions	Process tracking data, FCHVs attending the training, based on attendance logs
Implementation	Percentage of the health facilities implementing the minimum standard of program implementation.	At least 80% of the health facilities will implement a minimum standard for program implementation. Implementation will be a dichotomous measure (successful vs inadequate). A health facility will be defined as having successfully implemented the program if: (1) health facility in charge conducts monthly coaching and technical support at least 8 out of 12 months in a year; (2) 80% of FCHVs conduct first home visits to at least the 80% of the target clients; (3) 80% of FCHVs conduct at least one group meeting with at least 80% of the target clients; and, (4) 80% of FCHVs submit monthly reports at least 8 out of 12 months in an implementation year. As secondary outcomes, we will also: ● assess the extent to which each core component was implemented and ● report the percentage of health facilities implementing all core components. ● explore facilitators and barriers to implementation.	Healthcare worker survey, Process tracking data: including a monthly checklist documenting intervention component completed, and HTN patients' participation in them, to be collected by the health facility in charge during standing monthly meetings with FCHVs. Meeting observations and periodic reflection interviews will be used to explore fidelity and the facilitators and barriers.
Maintenance	Percentage of the health facility implementing a minimum standard to program implementation during the maintenance period	At least 80% of the health facilities will implement a minimum standard for program implementation during the maintenance period. Implementation will be a dichotomous measure (successful vs inadequate). A health facility will be coded as having successfully implemented the program if: [1] The health facility in charge conducts monthly coaching and technical support at least 8 out of 12 months in a year; (2) 80% of FCHVs conduct first home visits of at least 80% of the target clients in the maintenance year; (3) 80% of FCHVs conduct at least one group meeting with at least 80% of the target clients in the maintenance year, and (4) 80% of FCHV submit monthly reports at least 8 out of 12 months in an implementation year. As secondary outcomes, we will also: ● assess the extent to which each of the individual core components were being implemented during the implementation phase, and ● Examine the percentage of health facilities that implement all core components.	Healthcare worker survey and process tracking data, including a monthly checklist documenting intervention component completed and HTN patients' participation in them, will be collected by the health facility in charge during standing monthly meetings with FCHVs. FGDs and KIIs will be used to explore the maintenance facilitators and barriers, as well as sustainability.
Effectiveness outcomes			
(A) difference between the intervention and control group at 12 months in		(1) mean systolic and diastolic BP (mmHg); (2) proportion of patients aware of their HTN diagnosis; (3) proportion of patients with controlled BP (i.e., less than 140/90 mmHg; (4) low physical activity i.e., less than 600 MET- minute; (5) intake of five or more servings of fruits and vegetables per day; (6) adherent to hypertension medication; (7) quality of life scores	The interviewer administered quantitative baseline and endline survey
(B) multiple economic and financial outcomes:	(i) affordability (cost), (ii) cost-effectiveness (iii) equity, and (iv) scalability (measured by the difference in cost per patient of CHPC compared to standard of care).	Affordability: measured by total incremental costs of care in the study arms; Cost-effectiveness: measured by calculation of incremental cost-effectiveness ratios (ICERs) per person for per BP control and per QALY gained; Equity (from patient-perspective): Household economic impacts in the study are measured by financial and economic costs. Disaggregated analysis, based on gender, ethnicity, and socio-economic status, will be conducted to assess disparities among the study groups. Scalability: Scenario-based and sensitivity analysis for scaling up this intervention at local, provincial and national levels.	The interviewer administered quantitative survey, including: ● Baseline and endline patient surveys; ● Facility based survey; ● Costing instrument to calculate total and component costs; ● Process tracking system; ● Weekly time logs.

### 2.8 Data collection methods

**Participant** **interview**: At baseline and follow-up, trained RA will collect in-person data, using interviewer-administered surveys and objective measurements on Android tablet with the REDCap electronic data collection platform. The data collection will include (a) Sociodemographic data including age, gender, marital status, level of education, occupation using pre-tested standard questionnaire; (b) Lifestyle behaviors including fruit and vegetable intake using the diet quality questionnaire [32], physical activity using the global physical activity questionnaire [33], alcohol consumption, tobacco use, knowledge of hypertension [34], salt intake, and adherence to prescribed medication [35]; (c) Quality of life through EQ-5D-5L questionnaire.

**Anthropometric measurement:** We will measure weight using a calibrated digital weighing scale wth participants in light clothing, and height barefoot against a wall using a measuring tape scaled in centimeters.

**Blood pressure measurement:** Trained RA will measure blood pressure using a calibrated OMRON-16 digital instrument three times between intervals of at least 5 min with the participant sitting with back supported, feet flat, and arm supported at heart level [36]. The average of the three measurements will be used for analysis.

**Healthcare providers interview:** Trained RA will interview healthcare providers in 102 health facilities to assess the current HTN control efforts, including guidelines, equipment, facilities, human resources, and medicine availability, using a semi-structured pre-tested questionnaire.

**Process tracking:** The process tracking system will serve as a monitoring tool for program implementation and maintenance. FCHVs will record of group meetings and home visits, including schedules, BP readings, medication adherence, and referrals. During monthly health facility meetings, the facility in-charge will document agenda, challenges, and solutions. Research staff will attend monthly meetings and will also contact FCHVs either in person or by phone, and maintain field notes on observations related to the implementation process [37].

**Periodic reflection interviews:** We will conduct periodic reflections (PRs) with FCHVs and healthcare workers from intervention health facilities to capture evolving experiences, contextual changes, and adaptations [38]. We will purposively select 12- 24 health facilities (1 - 2 health facilities per municipality) to ensure a diversity by geography, community demographics, health facility characteristics, and patient volumes. One heathworker per selected facility and two FCHVs per facility will participate in 30-min interviews conducted every 2 to 3 months in conversational Nepali. PRs will explore implementation activities, alignment with routine work, barriers, facilitators, and stakeholder responses. Interviews will be iterative, allowing exploration of how barriers and facilitators change over time.

**Meeting observations:** We will observe monthly health facility meetings in selected intervention facilities, selected HTN group meetings, and home visits by FCHVs every two to three months. Observations will include diverse settings and participant characteristics. These observations will focus on understanding how the intervention is integrated into routine health facility activities, meeting dynamics, participation, and communication between healthcare workers and FCHVs. Meeting observations will be guided by a checklist. Data gathered will be recorded in the form of field notes.

**Focus group discussions (FGDs):** We will conduct 6-9 FGDs with purposefully selected FCHVs in intervention sites post-implementation and post-maintenance to capture diverse experiences. FCHVs will represent varied experience, proximity to health facilities, need for peer support, literacy levels, communication styles, and caste or religion. The FGDs will be conducted in Nepali in a private accessible setting. The discussion guide will build on rapid analysis of PR to explore barriers and facilitators, and the strategies to strengthen intervention implementation and maintenance.

**Key informant interviews (KIIs):** We will conduct KIIs with 12-16 key informants from federal and provincial MoH and municipal officials at two time points: post-implementation and post-maintenance to explore support for the program, barriers and facilitators, and leadership perspectives. Participants will be purposively selected for diverse insights. Interviews will assess integration of CHPC into government systems and progress toward sustainability, with iterative questioning to explore emerging themes.

#### 2.8.1 Power and sample size

The primary aim is to assess whether the implementation model meets acceptable rates, i.e. 80% of adoption, implementation, reach, and maintenance of the CHW-delivered HTN care**.** All 51 intervention health facilities will be included, providing 4.2% precision with a cluster size of 9 CHW per health facility and an intraclass correlation coefficient 0.04 [39]. To assess the clinical outcome, 3572 patients, 35 per clusters across 102 clusters will be enrolled. Assuming standard deviation at 17.75 mmHg for HTN [18], and intra-cluster coefficient (ICC) of 0.015 [40], with 10% loss to follow-up, the study has 90% power to detect a 3 mmHg systolic BP (primary clinical outcome) in a subsample of participants with uncontrolled hypertension, approximately 60% of the total. These calculations were computed using PASS 2024, version 24.0.2.8 [41].

### 2.9 Analysis plan

**Reach, Adoption, Implementation, and Maintenance:** We will assess adoption, implementation, reach and maintenance (Table 2) by calculating the proportion of facilities that achieve each component and all program components, with 95% confidence interval. Qualitative data from PRs, observations, KIIs and FGDs will be triangulated. PRs and observation-based data will be analzed using CFIR-based deductive rapid analysis [42] to identify barriers, facilitators, and the fidelity of the CHPC intervention at FCHV and health facility level. The primary analyst will document detailed notes, summarizing findings in matrices, and a secondary analyst will review recordings to refine the analysis. We will analyze FGDs and KIIs abductive analysis [43], using CFIR in NVivo 12.0.

**Clinical Effectiveness**: We will analyze the primary outcome, mean systolic BP at 12 months using intention-to-treat approach, with generalized estimation equation (GEE) with the identity link, exchangeable working correlation, and robust variance. If a substantial baseline imbalance occurs, we will conduct adjusted sensitivity analysis adjusted for age, marital status, education to assess the robustness of the primary analysis. We will also use ANCOVA for clustered data to conduct difference-in-difference analysis most efficiently [44]. In ‘per protocol’ analysis, we will exclude participant with fewer than 2 visits, no follow-up assessment, or post-randomization ineligibility. Causal inference probability weighting methods will also adjust for non-adherence to the protocol [45]. Secondary outcomes will also be analyzed using GEE with the identity link (for continuous variables) and the log link for binary variables with exchangeable correlation and robust variance. We will conduct stratified analyses to examine treatment effects by rural and urban areas, blood pressure control status at baseline, and biological sex.

**Costing:** We will calculate total implementation and maintenance costs by summing all components. We will examine associations between costs and key factors such urban/rural location, health facility catchment area, and FCHV-to-participant ratio using bivariate and multivariable analysis. We will also conduct budget impact analysis for assess the financial consequences of scaling the intervention at municipal level.

**Cost-effectiveness:** We will estimate incremental cost-effectiveness ratios (ICERs) per BP-controlled participant and per quality-adjusted life-year (QALY) gained based on WHO threshold for Nepal [46]. Economic evaluation will be done from the health system perspective with a discount rate of 3.5% [47]. We will conduct probabilistic and deterministic sensitivity analysis to test the robustness and assess the best- and worst-case scenarios, following Gold Commission guidelines [48] and the Bill & Melinda Gates Foundation [49].

**Equity:** We will collect patient survey data on out-of-pocket (OOP) costs and compare the OOP costs between CHPC participants and those the usual care. This will allow assessment of us to equity, by examining financial risk protection and differences in OPP costs across care models. Using extended cost-effectiveness method (ECEM) [50], we will assess distributional impacts by gender, ethnicity, and socio-economic status. The ECEA will estimate catastrophic health expenditure [51,52], defined as medically expenditure exceeding 10% of household income, using OOP cost survey data [53].

#### 2.9.1 Quality control and assurance

The data manager will review data weekly to identify missing and anomalous values. We will follow up with sites and participants to remedy identified data issues. We will pre‐test surveys and qualitative tools before deployment. We will document reasons for drops and monitor loss to follow up through monthly reports to the sites and Data Safety and Monitoring Board. We will summarize missing data and potential baseline imbalances and report analyses following CONSORT guidelines [54]. We will use inverse probability weighting (IPW) [55,56] and multiple imputations [57] to address loss to follow-up and missing covariates. For qualitative data, we will ensure quality through standardized guides, trained data collectors, audio-recorded interviews, accurate transcription checks, secure storage, reflexive team discussions, consistent coding, inter-coder reliability checks, and triangulation across data sources.

#### 2.9.2 Study management and oversight

The study is conducted through a multi-institutional collaboration between Yale School of Public Health, USA; Dhulikhel Hospital–Kathmandu University Hospital (DH-KUH), Nepal, and the Institute for Implementation Science and Health (IISH), Nepal. IISH leads field operations including data collection, field team management, digital data systems, logistics, and data management. DH-KUH provides clinical oversight, develops training materials, conducts monitoring and evaluation, and coordinates engagement with municipal, provincial, and federal government bodies. Yale School of Public Health ensures adherence to international standards for data quality and study protocol, with shared analysis. A Steering Committee including Ministry of Health representatives provides guidance in implementing the intervention within the government health system. An independent Data Safety Monitoring Board tracks and monitors safety and trial progress biannually.

## 3 Discussion

This paper presents the protocol of a community-based hypertension control trial to be conducted in Nepal. In the protocol, we have described the intervention strategies, planned their implementation, and detailed how we will assess the outcomes. HTN is a growing global public health concern. In Nepal, HTN management faces multiple challenges, including geography, shortages of health professionals, lack of effective primary health care level management and significant financial burden. This study addresses an urgent need for cost-effective, evidence-based HTN control strategies in low-resource settings.

This study will systematically facilitate and examine conducting and maintaining a contextually appropriate evidence-based FCHV-led HTN management intervention among adults in Nepal. While HTN control in Nepal has historically focused on health system-level interventions through training health workers in primary healthcare settings, CHPC will gather evidence on the effective implementation of a community-level HTN control. This focus on HTN control in the community will benefit patients with HTN along with apparently healthy adults, place HTN control on the community's agenda by focusing on community-based health advocates. By building capacity among health workers and FCHVs in Nepal, and embedding the program within their existing roles, we aim for high institutionalization within the primary health care system, with the potential for broad-based impact across the health system.

We have carefully considered potential challenges and proposed alternative solutions. Given the strong provincial MoH support, we expect a high proportion of health facilities to participate in the program. However, there is a small possibility that we could observe lower-than-expected levels of program adoption. In this event, we will turn to additional avenues to engage health facilities in charge and FCHVs, such as the use of community partners and direct relationships with municipalities.

If the CHPC has potential to demonstrate implementation performance, clinical effectiveness, and cost-effectiveness, it could provide a feasible pathway for scaling community-based hypertension management within Nepal's national health system. More broadly, the study will contribute to global implementation science by generating evidence on how task-shifting can strengthen NCD care delivery in LMICs. Lessons learned from this study may inform future community-based models for managing other chronic conditions such as diabetes and cardiovascular disease risk factors.

### 3.1 Strength and limitations

The primary strength of this study lies in its Hybrid Type III cluster-randomized design, which simultaneously evaluates clinical effectiveness and the real-world implementation process. By utilizing the health facility as the unit of randomization, the trial aligns with Nepal's existing primary healthcare infrastructure, increasing the potential for institutionalization and scalability. The use of a mixed-methods approach, guided by the RE-AIM and CFIR frameworks, provides a comprehensive understanding of implementation fidelity and contextual barriers. Furthermore, the inclusion of an Extended Cost-Effectiveness Analysis (ECEA) ensures that the findings offer actionable data for policymakers regarding the reduction of catastrophic health expenditures for rural populations.

However, the study has certain limitations. First, the trial uses non-blinded design due to then ature of the intervention, which may introduce performance bias among healthcare workers and participants. Second, implementation outcomes may vary across municipalities because of differences in capacity, geographic accesibility and community engagement. Third, although intervention is integrated within existing health systems, variations in FCHV motivation and local leadership support may incidence program fidelity and sustainability. Fourth, while the risk of cross-cluster contamination was formally assessed and mitigated through geographic buffering and restricted training, some spillover may occur during municipal-level administrative meetings. We will monitor this through qualitative probes in our periodic reflections and address it via a secondary per-protocol analysis if significant. Finally, the reliance on self-reported data for lifestyle behaviors may introduce social desirability bias.

### 3.2 Conclusions

At the conclusion of this study, we will have tested a scalable primary healthcare model for implementing and maintaining a community-based HTN control in Nepal. If successful, it will offer practical strategy for Nepal and other LMICs to mitigate the burden of HTN and contribute to guide scale up of interventions with Dissemination and Implementation (D&I) research literature.

## Ethics statement

The study has received formal ethics approval from two institutional bodies to ensure comprehensive oversight of both implementation and data management. The Ethical Review Board of the Nepal Health Research Council (NHRC) in Nepal granted approval on 14 February 2024 (Reg. No. 561/2023), covering all field-based study components, community screenings, and participant interactions across the 102 health facility sites in the Sindhupalchowk district. Additionally, the Yale Institutional Review Board (IRB) in the United States approved the study on 6 February 2024 (IRB #20000036053), focusing on data oversight, human subject protection, and analysis leadership. We have obtained permission for the study from provincial and local level stakeholders, including the Public Health Office in Sindhupalchowk. A support letter was also obtained from Nepal's Federal Ministry of Health and Population. All activities will be conducted in collaboration with these stakeholders and health facilities. Written and verbal consent will be sought before data collection from all participants, including patients with HTN, health care workers, the FCHVs, and federal, provincial, and municipal officials as relevant. The confidentiality of data obtained from the participants will be maintained throughout the study, ensuring protection of their identities from unauthorized parties and assurance of anonymity. Only the consent forms will contain the name and signature of the participants. Data will be collected in a private space at participants' houses or health facilities and a unique code will be assigned to the participants as identification numbers. The identifiers will be stored in a password-protected computer separately from the data, connected by an alphanumeric code. Only authorized study personnel, including the statistician and data manager, will have access to data and information in REDCap. All research related interaction, including phone calls and in-person meetings, will be conducted securely, and confidentiality will be maintained throughout the intervention. The research staff will be trained on ethical issues, including not sharing any information on study participants outside of the research team. No report, presentation, or publication will identify participants' information.

## Authors' contributions

AS, DT, DC, AH, DN, MD, RMK and DS designed the implementation trial. AS, SS and PS developed the first manuscript draft; all authors reviewed the protocol critically and provided feedback. DT, AS, AP, PS and SM were responsible for the trial registration and ethics approval. AS, AP, DT, DC and DS designed the protocol for randomization and statistical analysis. SS contributed to qualitative method design and analysis. DT, PS, SM, LP and RS will be responsible for the data collection. AS, SS, DRC, BP, AJ, PP and RS will be responsible for monitoring implementation of the trial.

## Trial status

Participant recruitment started in December 2024 and is projected to be completed by April 2026. Regarding intervention activities, all preparatory stages including orientation for provincial and local stakeholders and the development of training modules for Female Community Health Volunteers (FCHVs) have been successfully completed. While training sessions are underway, full field-level implementation of the Community-based HyPertension Control (CHPC) strategy is scheduled to begin in January 2025. The research team explicitly confirms that the trial is ongoing and that no primary or secondary outcome data have been accessed, cleaned, or subjected to any form of analysis.

## Funding

The study has received funding from the 10.13039/100026007National Bar Institute of 10.13039/100018696Health (10.13039/100000002NIH), USA (Project number: 1R01HL169421).

The funding agency has no role in the design of the study; and will not be involved in the collection, analysis, and interpretation of the data; or the writing of the manuscript.

## Declaration of competing interest

The authors declare that they have no known competing financial interests or personal relationships that could have appeared to influence the work reported in this paper.
